# Quality of Rehabilitation Services to Disabled in a Rural Community of Karnataka

**DOI:** 10.4103/0970-0218.42066

**Published:** 2008-07

**Authors:** S Ganesh Kumar, Acharya Das, Shashi Joyce Soans

**Affiliations:** Department of Community Medicine, Kasturba Medical College, Mangalore, Karnataka, India; 1 Department of Community Medicine, K.S. Hegde Medical College, Mangalore, Karnataka, India

## Introduction

Improving the quality of life of people with disabilities is a difficult and challenging task. World Health Organization estimates that 10% of the world's population has some kind of disability, and around 80% of the disabled population reside in rural areas. In developing countries, it was estimated that not more than 2%-3% of the disabled could benefit from rehabilitation services.(1)

Most authorities believe that disability in the community is a minor problem and does not need much of intervention. However, in reality, it is a social problem where the disabled population becomes a liability to the society. Alma Ata declaration in 1978 stated that a comprehensive primary healthcare should include promotive, preventive, curative and rehabilitative care. The major objective of community-based rehabilitation (CBR) is to ensure that people with disabilities are able to maximize their physical and mental abilities, have access to regular services and opportunities and achieve full social integration within their communities.(2)

There is paucity of facilities and services for the disabled in both governmental and non-governmental sectors. Moreover, such services are concentrated in the urban areas. The Government of India has taken several measures, including the Disability Act of 1995, for rehabilitation, and Non-Government Organizations too play a major role in this. In spite of these measures, disabled people still lack access to opportunities such as education, health, vocational guidance and employment apart from their emotional and psychosocial needs being neglected. Very few community-based studies had been conducted in India to understand the quality of rehabilitation services. Such studies have been useful tool in developing CBR programmes for the disabled. In view of the above context, the present study attempts to analyze activities of daily living (ADL) and quality of rehabilitation services provided to the disabled in a rural area of Karnataka.

## Materials and Methods

This was a community-based cross-sectional study carried out for a period of one year from January to December 2004. The study was conducted in four villages of the rural field practice area of a teaching institution, which covers a population of 45,000 in over 11 villages of a taluk in Karnataka state, India. The population covered by the four selected villages was 16,298. The sample size was estimated as 900, taking prevalence as 10% and precision of 20%. After adding non-response error of 10%, an additional 100 subjects were included. Thus, 1000 subjects of different age groups were selected for this study. Database of family particulars of each household was maintained in each village. Probability proportional to sampling technique was used to select the study sample from each village.

As part of the study, house-to-house visits were conducted for interviewing and examining all the individuals in the selected households with pre-tested questionnaire. The eligibility for membership in a household was defined as persons who are biologically related with other members and eating from a common kitchen. If a designated person could not be contacted or was not cooperative during two consecutive visits after the first, then the subject was considered as non-respondent. Disability was assessed as per the criteria laid down by WHO.(3) Mental disability was assessed by Indian Disability Evaluation and Assessment Scale (IDEAS) developed by the Rehabilitation Committee of Indian Psychiatric Society.

Various studies have used ADL for grading the severity of functional limitations. In this study, Modified Barthel Index (MBI) was used to measure ADL. It refers to the abilities needed for self-maintenance in the basic functions of personal hygiene, bathing self, feeding, toilet, stair climbing, dressing, bowel and bladder control and ambulation. The total score ranges from 0 to 100, based on the ability to perform each of the above tasks. Dependency level is classified as total (0-24), severe (25-49), moderate (50-74), mild (75-89) and minimal (90-99). Felt and received needs of the disabled or their families were assessed by interview technique with a focus on different types of intervention methods at the government and non-government levels. The instrument for needs assessment was designed on the lines of questionnaire taken from Action Aid India. The collected data were tabulated and analyzed using the Statistical Package for Social Sciences (SPSS) version 11.5 for Windows.

## Results

Out of 1000 subjects, 46 were non-respondents; 60 subjects were found to be disabled. The distribution of disability was as follows: mental (22), locomotor (17), speech (12), hearing (13) and visual (10). Majority (80%) of the disabled had a single disability.

Around one-third of the disabled (22) independently performed their ADL. Majority of them had mild hearing, speech and mental disability. Nearly one-fourth of the disabled had severe and total limitation on ADL, and most of them had locomotor disability [Table 1].

**Table 1:** Activities of daily living among disabled

Activities of daily living	Types of disabilities					Number of disabled (%)
		
	Visual	Hearing	Speech	Locomotor	Mental	
Independent	0	8	6	0	11	22 (36.7)
Minimal	7	4	2	0	6	16 (26.7)
Mild	0	0	1	3	1	4 (6.7)
Moderate	1	0	1	3	1	4 (6.7)
Severe	2	1	1	10	2	12 (20.0)
Totally disabled	0	0	1	1	1	2 (3.3)

Majority of the mentally disabled (17) were assessed, but only half of them received medical treatment. Although half of them expressed the need for counselling, very few (3) received it. Out of the three disabled felt for special schooling and scholarship, only two of them received it. Although 35%-75% of the disabled felt that they required vocational training, job placement and pension, only one accomplished this. None of the disabled received travel concession passes in spite of the fact that 16 of them felt the need for it [Table 2].

**Table 2:** Needs of the mentally disabled

Types of needs	*n* = 22	
	
	Felt (%)	Received (%)
Assessment	20 (90.9)	17 (77.3)
Medical/surgical treatment	17 (77.3)	11 (50)
Occupation therapy	12 (54.5)	0 (0)
Counselling	11 (50)	3 (13.6)
School	3 (13.6)	2 (9.1)
Scholarship	4 (18.2)	2 (9.1)
Vocational training	8 (36.4)	1 (4.5)
Job placement	10 (45.5)	1 (4.5)
Pension	17 (77.3)	1 (4.5)
Pass for travel	16 (72.7)	0 (0)

Only three visually disabled got medical/surgical treatment. Although majority (40%-100%) of the visually disabled felt that they required occupation therapy, aid/appliance, vocational training, job placement, pension and travel passes, none of them received any such facility. Only three hearing disabled received medical/surgical treatment and one in them received aid, scholarship and pension. However, none of them received vocational training, job placement or travel passes. Although eight (66.7%) of speech disabled felt the need for speech therapy, only four of them received it. Out of the three speech disabled who felt for special school and scholarship, only one received it. Majority (16) of the locomotor disabled were assessed, in which only eight (47.1%) received medical/surgical treatment. Although most (11) of them felt the need for physiotherapy intervention, only two (11.8%) received it. Only one of the locomotor disabled was sanctioned pension. None of them received occupation therapy, aid/appliance, vocational training, job placement and travel passes.

## Discussion

Our study demonstrated that majority of the disabled were able to do their ADL, and around one-fourth of the disabled required special care and attention. According to a recent report of National Sample Survey Organization, 60% of the disabled in the rural area were able to take self-care without any aid/appliances. Around 13% were observed to be severely disabled even with aid/appliances.(4)

The studies on the quality of rehabilitation services are relatively few. Our study showed that around 50% of the disabled received some kind of medical or surgical services, but other types of rehabilitation services were poor. Other studies found that only 2%-3% of the disabled have access to such services.(5) There is considerable need for the improvement of facilities, services and opportunities for the disabled.(6) Another study in rural Bangladesh showed that around 81% of the disabled had utilized some kind of healthcare, while more than half consulted unqualified practitioners of modern medicine.(7) A study conducted in Tamil Nadu found that 98% of the visually disabled did not use spectacles, and only 1.5% of them expressed the need for spectacles. Ninety-six percent of the hearing disabled did not use hearing aids. Only 3% of the disabled expressed the need for hearing and other aids like crutches, tricycles and callipers.(8) However, our study revealed that majority of the disabled felt the need for aids. According to our study, only 3% of the disabled were employed in contrast to 26% at the national level.(4) It shows that there is large scope for CBR for the disabled.

Received needs of the disabled could not be assessed because of feasibility constraints, and it required the involvement of specialists concerned. In cases of severe disability, parents or children of the disabled were interviewed to assess felt and received needs.

Disability is an important public health problem in our study area. There is a large gap between the services felt and received by the disabled in this part of the country. In the light of the above findings, it is concluded that the disabled in this area need community assistance. There is ample scope for CBR of the disabled.
